# Post-Cataract Surgery Iridodialysis Repair: A Simple Modified Ab Externo Technique

**DOI:** 10.7759/cureus.25676

**Published:** 2022-06-05

**Authors:** Madihah Mohd Lokman, Teck C Cheng, Jemaima Che Hamzah

**Affiliations:** 1 Department of Ophthalmology, Faculty of Medicine, Universiti Kebangsaan Malaysia, Kuala Lumpur, MYS

**Keywords:** ab externo, surgical technique, iridodialysis repair, complication, cataract surgery

## Abstract

Iridodialysis occurs less commonly due to ocular surgeries as compared to trauma. Several approaches to iridodialysis repair have been described in the literature. In this report, we describe a novel technique to treat iridodialysis that occurred due to complicated extracapsular cataract extraction (ECCE). This technique utilizes pre-existing ECCE wounds by making use of common materials found in the usual operation theaters and is relatively easy to master. Improved cosmetic appearance and visual acuity was the final outcome as demonstrated in our patient.

## Introduction

Iridodialysis is a condition in which the iris root is disinserted from the scleral spur [1]. It occurs most commonly secondary to trauma, and it is more frequently associated with blunt traumas compared to penetrating eye injuries. Iridodialysis can also occur due to intraocular surgeries. The prevalence of iridodialysis secondary to blunt trauma was reported to be 9.3-14% [2,3] as compared to 0.2% caused by cataract surgeries [4]. Small and superiorly located iridodialysis may be left untreated. However, a more inferiorly placed, larger, or symptomatic iridodialysis warrants surgical repair [5]. Patients with iridodialysis may report symptoms of glaring, photophobia, monocular diplopia, or reduced vision if the iris tissue covers the pupil. Cosmetically, it can lead to disfigured appearances in patients with the presence of corectopia or polycoria.

We describe a novel modified open chamber approach for repairing iridodialysis in a 65-year-old patient who sustained left eye iridodialysis secondary to a complicated extracapsular cataract extraction (ECCE) surgery. There was a moderately large iridodialysis spanning three clock hours superiorly. The decision for a surgical repair was made based on the worsening of left eye visual acuity postoperatively and exposure of the edge of the intraocular lens (IOL) at the periphery with polycoria.

## Case presentation

Surgical technique

**Figure 1:**
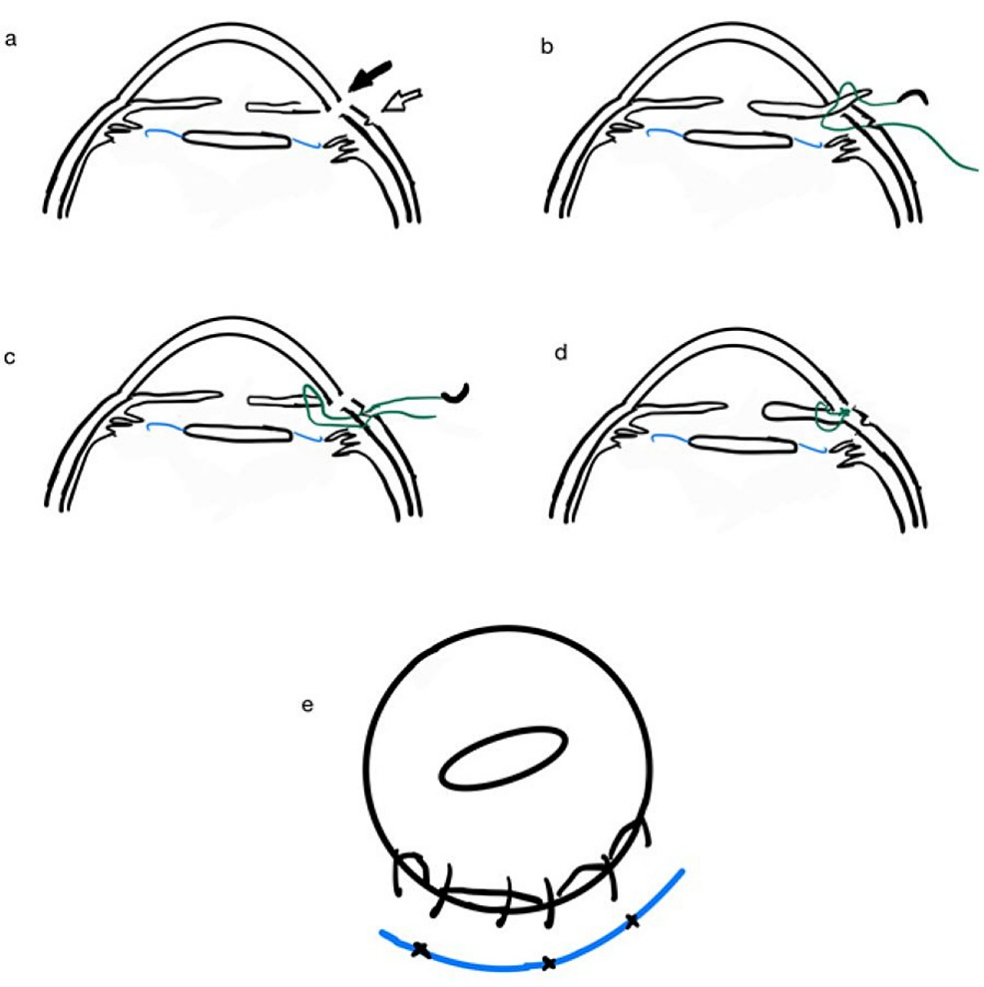
Surgical technique a) Under sub-Tenon’s anesthesia, a superior fornix-based conjunctival peritomy was made over the previous ECCE wound site (10-2 o'clock) (black arrow) and the anterior chamber was accessed through the same wound. A scleral groove (white arrow) was created 1.5 mm away from the limbus along the iridodialysis site. b) Single-armed 10/0 polypropylene suture was passed from the scleral groove into the anterior chamber (AC) to reach the peripheral iris. c) Interrupted mattress suture was placed between the sclera and peripheral iris tissue. A total of three interrupted mattress sutures were applied for this repair. d) Knots were placed hidden under the scleral groove. e) Final outcome of iridodialysis repair (surgeon’s view) ECCE: extracapsular cataract extraction

Under sub-Tenon’s anesthesia, we performed a superior fornix-based conjunctival peritomy along the iridodialysis site, from 10 to 1 o'clock. Next, we created a partial-thickness scleral groove parallel to the iridodialysis, 1.5 mm away from the limbus. Iris repair was initially attempted via closed chamber, ab interno technique in which a straight needle of 10/0 polypropylene suture was inserted 180˚ away from the iridodialysis site. The needle was advanced superiorly to the detached peripheral iris. However, the procedure was abandoned due to the difficulty in engaging the peripheral iris tissue. An open chamber approach was then carried out. Previous corneal limbal sutures from the ECCE wound were released in parts according to the site of repair. A single-armed curved needle of 10/0 polypropylene suture was advanced through the scleral groove at 12 o’clock and passed out externally from the anterior chamber (AC) through the limbal wound. Peripheral iris tissue was pulled out from the same wound using Colibri forceps. The suture was then passed through the peripheral iris from its posterior surface and again through its anterior surface in a mattress pattern. The needle from the AC was then directed out through the scleral groove from its inner surface. The knot was tied using a 3-1-1 method and then buried under the scleral groove. Subsequent interrupted mattress sutures were done at 11 and 1 o’clock. The limbal wound was closed with 10/0 Ethilon sutures and the conjunctiva was closed at 10 and 2 o’clock with 8/0 Vicryl sutures. The procedure was performed as illustrated in Figure 1.

Results

A 65-year-old female with multiple comorbidities had an ECCE performed for her left eye. She had been initially scheduled for phacoemulsification but the decision to convert to ECCE had been made due to an extensive breach of the anterior capsule during the main wound incision. During the surgery, the patient’s iris was floppy and kept prolapsing through the main wound. Iris reposition was performed multiple times, which resulted in iris atrophy and iridodialysis extending from 10 to 1 o’clock superiorly. Nevertheless, the cataract was successfully removed and IOL was inserted into the sulcus since the extension of the anterior capsule breach was failed to be identified. Unfortunately, she also sustained grade II hyphema due to intraoperative manipulation.

**Figure 2:**
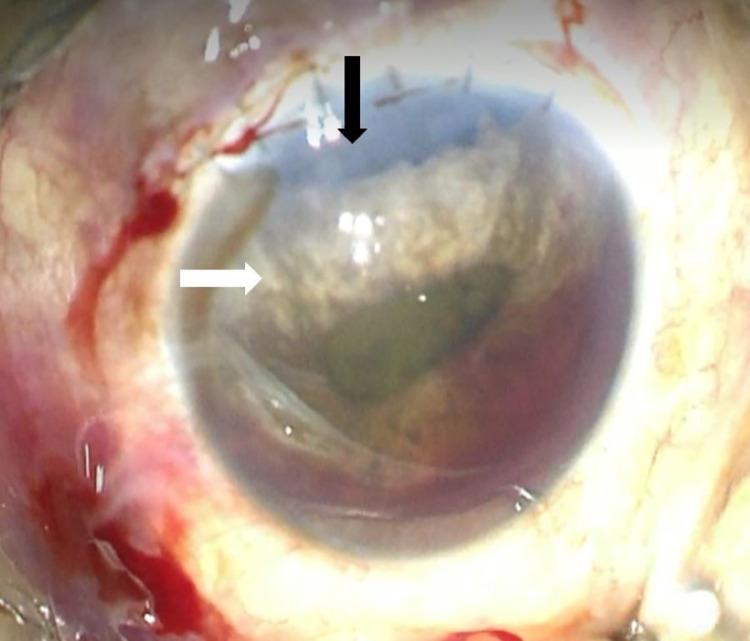
Iridodialysis The photograph shows the patient’s left eye prior to iridodialysis repair. Iridodialysis extends from 10 to 1 o’clock, exposing the peripheral IOL (black arrow). The superior half of the iris was atrophied (white arrow). The pupil was irregular secondary to the iridodialysis. There was also grade II hyphema present in the anterior chamber IOL: intraocular lens

At one week post-surgery, her best-corrected visual acuity was 6/12 and ocular findings were as shown in Figure 2. However, at two weeks postoperatively, the patient complained of decreased vision in the operated eye. Her vision dropped to 6/24 and slit-lamp examination showed minimal AC inflammation with the resolution of hyphema. A limbal wound leak evidenced by a positive Seidel’s test was seen at 11 o’clock.

**Figure 3:**
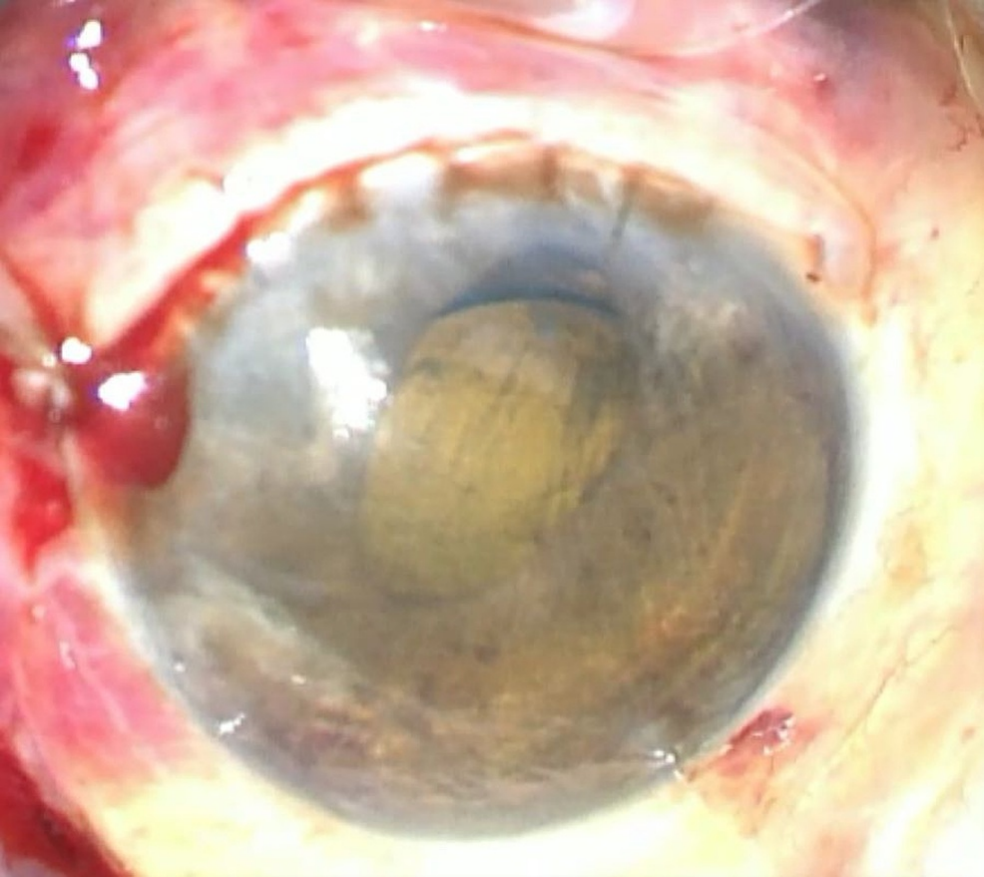
Post-iridodialysis repair Image immediately after iridodialysis repair; the pupil was rounder and more central with minor residual corectopia

Iridodialysis repair technique as previously described and limbal wound re-suturing was performed under the emergency setting. The pupil was more central and round immediately after iridodialysis repair as depicted in Figure 3. The vision improved to 6/9 at one month postoperatively.

## Discussion

The management of iridodialysis depends on several factors, which include the location and extent of the dialysis, and whether it has a physical or psychological impact on the patients [5,6]. Superior iridodialysis might not require repair as it can be covered by the upper eyelid. Small dialysis may be managed medically alone by cycloplegic eyedrops. Surgical repair is indicated if the iridodialysis is large, located more inferiorly, or gives rise to symptoms that reduce visual function [5], or if it is cosmetically unacceptable and impacts the patients psychologically [6]. For our patient, a surgical iridodialysis repair was performed due to reduced vision as the iris tissue was covering the visual axis.

The surgical technique for iridodialysis was first described by Key back in 1932 [7]. Ever since then, various surgical iris dialysis repair techniques have been described in the literature. Repair techniques can be broadly classified into open and closed chamber techniques. Open chamber techniques utilize limbal corneal incision or scleral incision to gain access into AC [8,9], while closed chamber techniques utilize the passage of the needle through the anterior chamber to gain access to the iridodialysis site [10,11]. Open chamber techniques have been associated with an increased risk of infection due to larger wounds. Closed chamber methods have a better safety profile, but the manipulation is more challenging.

The technique we described was a modified open chamber iridodialysis repair. We utilized the previous corneal incision made during ECCE to gain access to the AC. For the surgeon, this obviated the need of performing extra incisions. It also was cost-effective as we only utilized one single-armed 10/0 polypropylene suture for the iridodialysis repair and no special or expensive instruments were required. The instruments used are commonly found in the usual operating theaters. This technique provides visibility of the peripheral iris throughout the whole procedure.

Several repair techniques such as the sewing machine technique [12] and knotless technique [13] limit the visibility of the edges of the peripheral iris after the passing of a few initial sutures, especially during the passage of the last suture. Poor visibility may result in the passage of suture through the thicker peripheral iris, which may cause corectopia. Using our technique, the peripheral iris tissue was visible throughout the whole operation. On the other hand, the knotless technique might have a problem with wound stability.

Some surgeons prefer to employ continuous suture placements. The advantage of this suture technique is that there will be a firm attachment of the detached peripheral iris to the sclera, but it also might lead to angle closure. We avoided this risk by placing interrupted mattress sutures that prevent iris clumping onto the trabecular meshwork. Knots were hidden under the scleral groove in this technique to minimize the risk of infection and reduce postoperative discomfort. Another method described to tackle these issues involves the placement of knots in the AC [14]. However, this requires an initial incision from the inner surface of the sclera before going in again to the AC. The incision might be more difficult, and the needle might become blunt after multiple incisions through the full-thickness sclera. The larger incision with multiple sutures used in this technique may also contribute to postoperative astigmatism and increase the risks of suture-related complications, such as infection.

Closed chamber techniques have a steeper learning curve and require superior surgical dexterity. Passing of needle through the AC also poses the risk of trauma to the overlying cornea or underlying anterior lens capsule if it is still intact, which is typical in an eye with blunt trauma.

## Conclusions

There are several open and closed chamber iridodialysis repair techniques described in the literature, and the choice of technique can be based on the surgeon’s preference and experience as well as the etiology of the iridodialysis. We believe that this technique will be especially beneficial in iridodialysis cases that occur as a complication of an intraocular surgery as we can make use of the incisions that were already made during the previous surgery.

## References

[1] Kumar S, Miller D, Atebara N, Blance E (1990). A quantitative animal model of traumatic iridodialysis. Acta Ophthalmol (Copenh).

[2] Ulagantheran V, Ahmad Fauzi MS, Reddy SC (2010). Hyphema due to blunt injury: a review of 118 patients. Int J Ophthalmol.

[3] Khan-Farooqi HR, Chiranand P, Edelstein SL (2010). Epidemiology and outcome of traumatic hyphema: a retrospective case series. Invest Ophthalmol Vis Sci.

[4] Sangameswaran RP, Verma GK, Raghavan N, Joseph J, Sivaprakasam M (2016). Cataract surgery in mobile eye surgical unit: Safe and viable alternative. Indian J Ophthalmol.

[5] Balamurugan R, Gupta PC, Sharma VK, Khurana S, Ram J (2020). Alternate iris bypass technique of iridodialysis repair. Indian J Ophthalmol.

[6] Pandav SS, Gupta PC, Singh RR, Das K, Kaushik S, Raj S, Ram J (2016). Cobbler's technique for iridodialysis repair. Middle East Afr J Ophthalmol.

[7] Key BW (1933). Concerning iridodialysis as a clinical entity, its surgical treatment: report of cases. Trans Am Ophthalmol Soc.

[8] McCannel MA (1976). A retrievable suture idea for anterior uveal problems. Ophthalmic Surg.

[9] Paton D, Craig J (1973). Management of iridodialysis. Ophthalmic Surg.

[10] Nunziata BR (1993). Repair of iridodialysis using a 17-millimeter straight needle. Ophthalmic Surg.

[11] Wachler BB, Krueger RR (1996). Double-armed McCannell suture for repair of traumatic iridodialysis. Am J Ophthalmol.

[12] Ravi Kumar KV (2018). Modified sewing machine technique for iridodialysis repair, intraocular lens relocation, iris coloboma repair, Cionni ring fixation, and scleral-fixated intraocular lens. Indian J Ophthalmol.

[13] Voykov B (2016). Knotless technique for iridodialysis repair. Clin Exp Ophthalmol.

[14] Ozdek S, Ozmen M (2009). A simple surgical technique for repair of iridodialysis. Turk J Med Sci.

